# Sex-Specific Lipid Profiles and Flavor Volatiles in Giant Salamander (*Andrias davidianus*) Tails Revealed by Lipidomics and GC-IMS

**DOI:** 10.3390/foods13193048

**Published:** 2024-09-25

**Authors:** Shibo Zhao, Jinghong Yu, Linjie Xi, Xiangdong Kong, Jinjin Pei, Pengfei Jiang, Ruichang Gao, Wengang Jin

**Affiliations:** 1 Qinba State Key Laboratory of Biological Resource and Ecological Environment (Incubation), Collaborative Innovation Center of Bio-Resource in Qinba Mountain Area, Shaanxi University of Technology, Hanzhong 723001, China; zsb20220225@gmail.com (S.Z.); yujinghong2000@163.com (J.Y.); xilinjie@snut.edu.cn (L.X.); 18700578289@163.com (X.K.); xnjinjinpei@163.com (J.P.); 2 Key Laboratory of Bio-Resources of Shaanxi Province, School of Bioscience and Engineering, Shaanxi University of Technology, Hanzhong 723001, China; 3 SKL of Marine Food Processing and Safety Control, National Engineering Research Center of Seafood, School of Food Science and Technology, Dalian Polytechnic University, Dalian 116034, China; jiangpf@dlpu.edu.cn; 4 School of Food and Biological Engineering, Jiangsu University, Zhenjiang 212013, China

**Keywords:** lipidomics, gender, differential lipids, volatile organic compounds, giant salamander

## Abstract

To elucidate the relationships between lipid components and odor traits, this study comparatively characterized the distinct lipid compositions and flavor volatiles in giant salamander tails of different sexes via mass-spectrometry-based lipidomics and GC-IMS. A total of 3145 fat metabolites were detected in male and female giant salamander tails, with the largest contributors being triglycerides (TGs, 840) and phosphatidylcholines (PCs, 383). Notably, the contents of PCs and TGs were greater in female tails than in male tails, and the levels of eicosapentaenoic acid (EPA) and docosahexaenoic acid (DHA) were also greater in the female group. Additionally, a total of 45 volatile components were detected, namely, 14 aldehydes, 14 alcohols, 9 ketones, 3 acids, 3 esters, 1 ether, and 1 amine. Alcohols (29.96% to 34.85%) and aldehydes (21.07% to 22.75%) were the predominant volatiles. Multivariate statistical analysis revealed 22 key differential fats and 26 differential odor substances as distinguishing labels between sexes. Correlation analysis revealed that the concentrations of triethylamine, dimethyl sulfide, ethanol-D, and 3-methyl butanal-D were significantly positively correlated with the concentrations of diglyceride (DG) (26:6e), cardiolipin (CL) (59:4), acylcarnitine (AcCa) (22:4), and triglyceride (TG) (52:10) (*p* < 0.01). Threefold cross-validation revealed that the prediction accuracies of these differential lipids and volatile compounds for sex recognition via the random forest model were 100%. These findings might not only provide insight into the effects of sexes on the lipid and volatile profiles of giant salamander tails but also provide clues for their gender recognition.

## 1. Introduction

As a living fossil and dioecious amphibian species, the giant salamander (*Andrias davidianus*) has been extensively farmed in China for its nutritional and medicinal value [1]. To date, the nutritional profiles of giant salamander [2,3], bioactive peptides [4,5], collagen/gelatin [6,7], ready-to-eat products [8,9], segmentation processing, storage, and preservation techniques [10] have been widely documented. Tail tissue in giant salamanders is underutilized because of its high fat content and fishy odor [11,12]. The lipids in giant salamanders are predominantly found in the tails, accounting for 13.88% of the body, with a crude fat content of 12.10%. Compared with fish oils, giant salamander fats present greater proportions of unsaturated and polyunsaturated fatty acids (PUFAs), especially eicosapentaenoic acid (EPA), docosahexaenoic acid (DHA), and linoleic acid [13,14].

Several investigations have shown that alterations in lipid profiles might influence the taste, appearance, and mouthfeel of animal-derived commodities [15,16]. During the processing and preservation of meat, lipase and phospholipase enzymes facilitate the hydrolysis of triacylglycerols and phospholipids, producing excess free fatty acids (FFAs). These FFAs are then oxidized to produce volatile compounds, including aldehydes, alcohols, and esters [17]. These volatile compounds, which have high volatility and low odor thresholds, are essential to meat flavor. Additionally, fat-soluble volatile constituents may accumulate within lipids and be released under appropriate conditions [18].

Lipidomics is an emerging tool that systematically examines the entire cellular lipid content, elucidating the mechanisms by which lipids participate in various biological processes, and facilitating further research into the complexity and diversity of food lipids [19,20]. Currently, gas chromatography-mass spectrometry (GC-MS) and liquid chromatography-mass spectrometry (LC-MS) are the primary tools widely employed for lipidomic analysis. LC-MS-based lipidomics offers a more comprehensive depiction of lipid molecule structures, making it the preferred method for lipid identification and quantification [15].

Additionally, the detection of flavor volatiles or odor compounds in food products has gained widespread attention. GC-MS and gas chromatography-ion mobility spectrometry (GC-IMS) are commonly employed technologies for analyzing odors or volatile flavors. Compared to the traditional GC-MS technique, GC-IMS offers significant advantages and promising applications, particularly in terms of speed, subtle differentiation, and corridor visualization, all without a complicated sample extraction process [21]. These technologies enable more accurate and detailed analysis of lipids and odor compounds in giant salamanders, facilitating the exploration of the associations between lipids and odor substances.

Numerous studies have validated the interplay between fat composition and odor substances in food products through lipidomics and volatile flavor analysis. For instance, Zhou et al. [22] characterized the key lipids responsible for odor formation in crayfish meat subjected to various heat treatments via lipidomics and volatile flavor characterization. Similarly, Zheng et al. [15] explored the influence of curing agents on fat compositions and odor substances in ham products via lipidomics and GC-IMS. These studies demonstrate the combined application of lipidomics and volatile flavor analysis for the characterization of lipids and odor profiles in various food products.

As giant salamanders are dioecious aquatic animals, it is challenging for consumers to distinguish their gender accurately in markets and artificial farm settings [23,24]. Research has shown that many animals exhibit sex-specific odors [25,26]. Additionally, different tissues of aquatic animals present varying levels of protein and lipids [27]. Consequently, the odor characteristics of male and female giant salamanders may differ due to variations in lipid accumulation. Lipid biomarkers are associated with sex, and volatile compound markers can be used for sex classification [28,29]. It is possible to achieve sex identification through lipid and volatile compound composition. For example, Fu et al. [30] studied *Crassostrea gigas* and reported that there are certain differences in gender-related odors. Similarly, studies on other animals such as abalones [31] and crabs [32] have confirmed sex-specific odors and lipids. Therefore, there are likely sex differences in the lipid composition and odor of giant salamanders.

Our previous studies investigated the quality characteristics and volatile flavors of jerky and fried meatballs from giant salamanders [8,9]. However, little information on the lipids and odor profiles of giant salamander tails according to sex has been reported. Therefore, the objectives of this study were to explore the differences in lipid and odor profiles between female and male giant salamander tails based on lipidomics and GC-IMS and to elucidate the relationships between lipids and odor substances. Moreover, the feasibility of the use of different lipids and odor chemicals for the sex prediction of giant salamanders was also investigated.

## 2. Materials and Methods

### 2.1. Material and Reagents

Six adult male giant salamanders (weighing 4.56 ± 0.26 kg) and six adult female giant salamanders (weighing 4.25 ± 0.31 kg) were selected by an experienced technical farmer and purchased from Hanzhong Weidani Aquaculture Co. Ltd. (Hanzhong, China). The slaughtering included quick stunning of the giant salamanders, bloodletting, and cutting up according to industrial practices [7]. Then, the tail tissues from both genders were chilled on ice and immediately transported to the Joint Lab for Bioscience Resources at Shaanxi University of Technology (Hanzhong, China).

Acetonitrile, isopropanol, and methanol were purchased from Thermo Fisher (Pittsburgh, PA, USA). Isotopic internal standards were purchased from Avanti Polar Lipids, Inc. (Alabaster, AL, USA); and formic acid (DIMKA) and ammonium formate (Honeywell Fluka) were obtained from the USA. SPLASH™. Other chemicals used for lipidomic research were of the highest analytical grade and were obtained from Sigma-Aldrich (Shanghai, China). Standard n-ketones were procured from Guoyao Chemical Co., Ltd. (Shanghai, China).

### 2.2. Arrangement of Tail Samples

The raw giant salamander tails were initially cleaned with running water, sliced, and ground separately. The samples were labeled male tails (MTs) and female tails (FTs) and then stored at −80 °C (for less than 48 h) prior to subsequent analysis to minimize lipid oxidation, which was consistent with our previous experimental preservation methods [33].

### 2.3. Lipidomics Quantitation

Six adult male giant salamanders and six adult female giant salamanders were used for lipidomic quantitative analysis. Tail samples from each individual were taken, with six replicates per sex. Lipidomic quantification of giant salamander tail samples was performed via a modified UHPLC-MS method based on Zheng et al. [15] with slight modifications. For lipid extraction, 100 mg of lyophilized tail samples from each sex were collected and mixed with 200 μL of water and 20 μL of an inner fat reference mixture. This mixture was agitated with 800 μL of MTBE, followed by the addition of 240 μL of precooled methanol. The samples were sonicated in a low-temperature water bath for 15 min, left at ambient temperature for 30 min, and then centrifuged at 14,000× *g* for 15 min at 10 °C. The upper organic phase was pooled and dried under nitrogen. For mass spectrometry analysis, the dried phase was reconstituted with 200 μL of a 90% isopropanol/acetonitrile solution and thoroughly vortexed, and then 90 μL of this reconstituted mixture was centrifuged at 14,000× *g* for 15 min at 10 °C. The supernatant was used for injection analysis. Moreover, equal amounts of samples from each group were combined to create a quality control (QC) sample, interspersed throughout the testing process of the samples to ensure system stability throughout the entire experimental process.

Lipid separation was carried out by a UHPLC Nexera LC-30A ultra-high-performance liquid chromatography system equipped with a C18 column (1.7 µm, 2.1 mm × 100 mm, Waters), according to Zhou et al. [22]. The column temperature was maintained at 45 °C and the flow rate was set to 300 μL/min. The gradient elution program was as follows: from 0 to 2 min, solvent B was maintained at 30%; from 2 to 25 min, solvent B linearly increased from 30% to 100%; and from 25 to 35 min, solvent B was maintained at 30%. During the analysis, the samples were kept in an autosampler at 10 °C. Detection was conducted via electrospray ionization (ESI) in both positive and negative ion modes. Following isolation by UHPLC(Nexera LC-30A, Shimadzu Corporation, Kyoto, Japan), the samples were analyzed using a Q Exactive series mass spectrometer (Thermo Scientific™, Waltham, MS, USA).

### 2.4. GC-IMS Quantitation of VOCs

Odor component detection in the tail samples of male and female giant salamanders was conducted via GC-IMS. The tail samples were homogenized with a tissue disperser, and 3.0 g of each sample was accurately weighed and placed into 20.0 mL headspace vials. The vials were then maintained at 60 °C for 20 min. Each sample was analyzed in six parallel groups. The detailed instrument parameters for quantitation were consistent with the conditions and parameters published previously [14].

### 2.5. Data Statistics

The data from the tests were initially organized via Excel, and the mean values ± standard deviations (*n* = 6) of the flavor components and lipid indicators were reported. *t*-tests and Pearson correlation analyses were subsequently conducted using SPSS 27.0 software. Volatile organic compounds (VOCs) were characterized via the NIST 2014 and IMS databases. Percentage plots and bar charts were created using Origin 2022. The data were then uploaded to MetaboAnalyst 6.0 for PLS-DA, PCA, and VIP calculations. Correlation heatmaps were generated using the pheatmap function in RStudio (psych, ggplot2, and ggsignif packages) and an online website (https://omicsolution.org/wkomics/main, accessed on 21 May 2024). Finally, the random forest method and threefold cross-validation were performed in Python version 3.12.

## 3. Results and Discussion

### 3.1. Lipid Compound Analysis in Giant Salamander Tails of Different Sexes

#### 3.1.1. Lipid Components of Giant Salamander Tails

**Figure 1:**
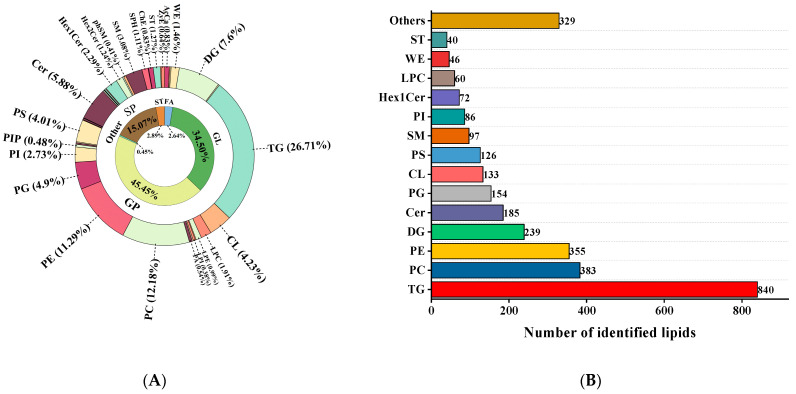
Overall lipid profiles of giant salamander tails of different sexes through lipidomic analysis. (**A**) The percentages of major and subclasses of lipids; (**B**) the number of all lipid species identified. The abbreviations in the figure are as follows: AcCa, acylcarnitine; Cer, ceramides; ChE, cholesterol ester; CL, cardiolipin; DG, diglyceride; Hex1Cer, monohexosyl ceramide; Hex2Cer, dihexosyl ceramide; LPC, lysophosphatidylcholine; LPE, lysophosphatidylethanolamine; LPI, lysophosphatidylinositol; PA, phosphatidic acid; PC, phosphatidylcholine; PE, phosphatidylethanolamine; PG, phosphatidylglycerol; phSM, phytosphingosine; PI, phosphatidylinositol; PIP, phosphatidylinositol(4)phosphate; PS, phosphatidylserine; SM, sphingomyelin; SPH, sphingosine/sphingosine bases; ST, sterol lipids; TG, triglyceride; WE, wax esters; ZyE, zymosterol.

A total of 2171 and 974 fat substances, respectively, were detected in giant salamander tails of different sexes via UHPLC-QE-MS in positive and negative ion modes (Figure 1A), corresponding to 44.45% glycerophospholipids (GPs), 34.50% glycerolipids (GLs), 15.07% sphingolipids (SPs), 2.98% sterol lipids (STs), 2.64% fatty acyl (FA), and 0.45% other lipids including glucosylsphingosine (SoG1), and prenol lipids (PR). This statistical method is based on categorizing lipids by type, and it identifies GPs and GLs as the main lipid categories. GPs are a major constituent of biological membranes and are involved in various cellular processes, including signal transduction, regulation of protein activity, and transport processes. GPs can readily serve as a substrate for the generation of odor substances [34]. Through lipidomics, Mi et al. [35] also studied the lipid profiles of different age and sex groups of Taihe silky fowl through lipidomics and reported that the prevailing lipids were GPs and GLs, which was in line with the present results.

As illustrated in Figure 1B, these lipid compounds may be further categorized into 44 subclasses, with triglycerides (TGs) being the most abundant subclass, comprising a total of 840 species. TG is the most crucial form of lipid in animals, and its breakdown into free fatty acids is crucial for flavor formation [15]. There were 383 phosphatidylcholines (PCs), 355 phosphatidylethanolamines (PEs), 239 diacylglycerols (DGs), 185 ceramides (Cers), 154 phosphatidylglycerols (PGs), 133 cardiolipins (CLs), 126 phosphatidylserines (PSs), 97 sphingomyelins (SMs), 86 phosphatidylinositols (PIs), 72 dihexosyl ceramides (Hex2Cers), 60 lysophosphatidylcholines (LPCs), 46 wax esters (WEs), 40 sterol lipids (STs), and 329 others. Lipids present in giant salamander tails predominantly consist of significant quantities of TGs, PCs, and PEs (Figure 1A). We found that the quantities of PCs and TGs in female tails (FTs) were substantially greater than those in male tails (MTs), indicating that the contents of PCs and TGs in the tails of female giant salamanders were greater than those in males. TGs constitute the primary component of fats in both plant and animal sources and serve as the main constituent for the storage of body fat in humans [36]. The oxidation of PCs to yield an abundance of volatile flavor compounds imparts a distinctive odor to giant salamander tails. Research has indicated that substantial amounts of PCs and PEs can play pivotal roles in quality deterioration and are considered key lipid metabolism products in the storage process of tuna [37]. Moreover, the composition and types of PCs and PEs are closely linked to the freshness of Atlantic salmon fillets [38]. Liu et al. [39] also demonstrated the lipid components in roasted pork through lipidomic analysis and reported that PCs, PEs, and TGs were the most predominant. The levels of DHA and EPA were greater in the female group than in the male group (Figure S1). The reason for this difference may be due to metabolic variations in giant salamanders of different sexes, and sex hormones may influence the enzymatic synthesis pathways of related unsaturated fatty acids (UFAs) [28,29].

#### 3.1.2. Multivariate Statistical Analysis of Lipid Compounds in Giant Salamander Tails

**Figure 2:**
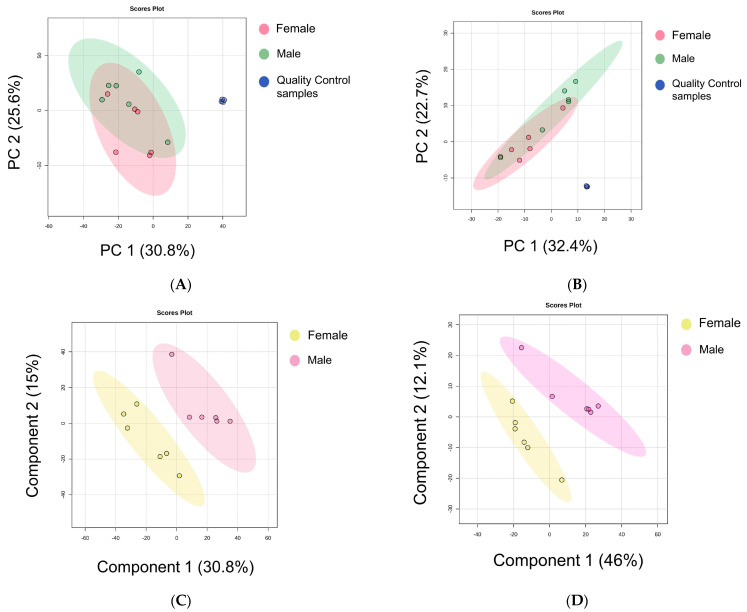
PCA score plots of test and quality control (QC) samples in positive (**A**) and negative (**B**) modes, respectively. PLS-DA scores of test samples in positive (**C**) and negative (**D**) ion modes in giant salamander tails of different sexes.

Principal component analysis (PCA) is an unsupervised learning tool that employs linear dimensionality reduction techniques for data analysis, visualization, and preprocessing. Its objective is to achieve a linear transformation that minimizes the error while converting data into a lower-dimensional space [30]. The PCA plot did not show differences among the lipid metabolites in the giant salamander tails of different sexes, as depicted in Figure 2A,B. The sum contribution percentages of the initial two main factors were 56.4% (PC1 30.8% and PC2 25.6%) and 55.1% (PC1 32.4% and PC2 22.7%) in the positive and negative modes, respectively. Both PCA methods (positive and negative) failed to effectively discriminate the lipid components within the giant salamander tails of different sexes. The quality control (QC) sample is used not only to assess the instrument status before sample introduction and to equilibrate the chromatography-mass spectrometry system but also interspersed among the test samples during the analysis process to evaluate the system’s stability throughout the experimental procedure [40]. The PCA plot revealed that the QC samples were tightly clustered, indicating that the experiment had good reproducibility.

Yang et al. [41] noted better sample discriminative effects of supervised methods when the PCA model failed. As unsupervised PCA plots did not differentiate between samples (Figure 2A,B), supervised partial least squares-discriminant analysis (PLS-DA) models were further constructed, which can effectively avoid the drawbacks encountered in unsupervised PCA. Figure 2C,D depict the PLS-DA analysis of lipid compounds identified in giant salamander tails of different sexes. The contributions of PLS-DA in the positive and negative ion modes were 45.8% and 58.1%. Moreover, supervised PLS-DA models were generated for each cohort, revealing a clear inclination toward group differentiation. The cross-validation outcomes of PLS-DA indicated *R*^2^ = 0.65438 and *Q*^2^ = 0.22054 in positive ion mode (Figure 2C) and *R*^2^ = 0.99958 and *Q*^2^ = 0.45523 in negative ion mode (Figure 2D), suggesting the commendable classification predictive capacity and consistency of both models. A similar lipidomic study using the PLS-DA model in mackerel at different processing stages was also reported by Liu et al. [42].

#### 3.1.3. Identification of Key Lipids

**Figure 3:**
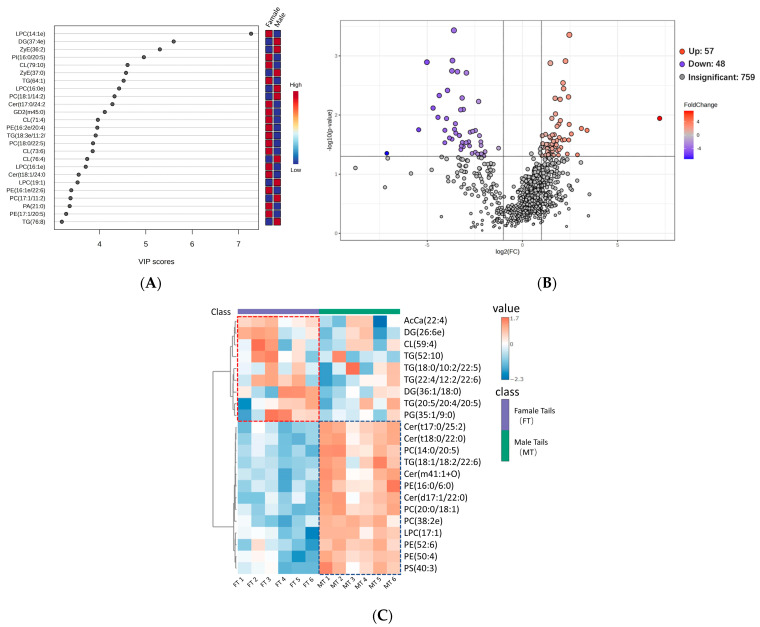
The VIP score plots of PLS-DA for the top 25 lipid components detected in giant salamander tails of different sexes (**A**). Volcano plots of the lipid components detected in giant salamander tails of different sexes (**B**). Heatmap of differential lipids (VIP ≥ 1, *p* < 0.01, FC > 2, or FC < 0.5, FDR < 0.05) (**C**). AcCa, acylcarnitine; Cer, ceramides; CL, cardiolipin; DG, diglyceride; GD2, disialo dihexosyl ceramide; Hex1Cer, monohexosyl ceramide; Hex2Cer, dihexosyl ceramide; LPC, lysophosphatidylcholine; LPE, lysophosphatidylethanolamine; LPI, lysophosphatidylinositol; PA, phosphatidic acid; PC, phosphatidylcholine; PE, phosphatidylethanolamine; PG, phosphatidylglycerol; phSM, phytosphingosine; PI, phosphatidylinositol; PS, phosphatidylserine; SM, sphingomyelin; SPH, sphingosine/sphingosine bases; ST, sterol lipids; TG, triglyceride; ZyE, zymosterol.

On the basis of a variable importance projection (VIP) ≥ 1 and *p* < 0.05, a total of 864 lipids in giant salamander tails were chosen as differentially abundant lipids (DALs). The top 25 DALs are illustrated in Figure 3A. The VIP values of lysophosphatidylcholine (LPC) (14:1e), diglyceride (DG) (37:4e), zymosterol (ZyE) (36:2), phosphatidylinositol (PI) (16:0/20:5), and cardiolipin (CL) (79:10) are relatively high. LPC is a phospholipid with choline as the head group and is also a member of the phosphatidylcholine group, a brownish-yellow lipid substance present in animal tissues. The synthesis of DG is initiated by glycerol-3-phosphate and typically takes place in the cytoplasm of tails or adipose tissue cells. PI comprises a group of lipids composed of a phosphate group, two fatty acid chains, and one inositol molecule. These compounds belong to the phosphatidyl glyceride class. Generally, phosphatidylinositols constitute a small proportion of the cytosolic side of eukaryotic cell membranes. CL is a distinct phospholipid synthesized and located in the inner mitochondrial membrane (IMM). CL plays a pivotal role in numerous reactions and processes related to mitochondrial function and dynamics [20].

Lipids containing unsaturated double bonds, such as CL (79:10), DG (37:4e), TG (76:8), and TG (18:3e/11:2/11:2), are closely associated with the flavor of meat products. Some studies have suggested that UFAs in fish are preferable to saturated fats found in fatty meat and lard. Fish oil can provide abundant long-chain n-3 polyunsaturated fatty acids [43]. Compared with the TG form, the PL form of n-3 UFAs has drawn widespread attention for its superior bioavailability, high tissue delivery capacity, and significant health-promoting functions linked to cardiovascular diseases [40,44]. Phospholipids, which contain phosphoric acid, can be further classified into GPs and SPa. Giant salamander tails contain a significant number of UFAs in the PL form, which implies potential prospects for the development of functional lipids.

Using the criteria of VIP ≥ 1, *p* < 0.05, and fold change (FC) > 2, or <0.5, DALs were identified and selected, as shown in Table S1. In Figure 3B, the volcano plot illustrates upregulated or downregulated differential lipids, where orange represents upregulated lipids, purple represents downregulated lipids, and gray indicates no significant change. Elevated VIP and decreased *p*-values indicate notable distinctions in the target lipids between the two sample sets. A total of 57 upregulated lipids and 48 downregulated lipids were detected within the male tail group compared with the female tail group (Figure 3B). Among the 57 upregulated lipids, Cer, Hex2cer, and ZyE were unique. Among the 48 downregulated lipids, AcCa, monohexosyl ceramide (Hex1Cer), SM, and CL were unique, whereas other lipids (such as DG, PC, PE, PG, and TG) tended to be upregulated or downregulated in different groups. In a study by Wang et al. [19], VIP ≥ 1 derived from the PLS-DA model, as well as *p* < 0.05 and FC ≥ 1.2 or ≤0.83), was utilized to discern DALs in 40-day stored shrimp and 0-day unstored shrimp samples. In another study conducted by Mi et al. [35], an investigation of the lipid composition of chickens of different sexes revealed significant distinctions in 11 lipid components between male and female groups. These findings suggest that sex can contribute to alterations in the tissue lipid composition of animal products.

Next, from the 105 lipid categories identified in the previous step (including 57 upregulated and 48 downregulated), we further filtered out lipids with significant differences on the following criteria: VIP ≥ 1, false discovery rate (FDR) < 0.05, *p* < 0.01, and fold change (FC) > 2, or <0.5. A total of 22 differential lipids were chosen from the 105 lipids for further correlation analysis. A heatmap of the 22 differential lipids was further created to visually contrast notably distinct lipid subtypes in giant salamander tails of different sexes. As illustrated in Figure 3C, lipids within the red box presented relatively high levels in the female group, including AcCa (22:4), DG (26:6e), TG (22:4/12:2/22:6), and PG (35:1/9:0), whereas lipids within the blue box presented relatively high levels in the male group, including Cer (t17:0/25:2), Cer (t18:0/22:0), PC (14:0/20:5), TG (18:1/18:2/22:6), Cer (m41:1 + O), PE (16:0/6:0), Cer (d17:1/22:0), PC (20:0/18:1), PC (38:2e), LPC (17:1), PE (52:6), PE (50:4), and PS (40:3). Individual color differences within groups may be due to experimental errors (Figure 3C).

The DHA present in TG (22:6) has been demonstrated to lower the risk of heart disease, prevent hyperactivity disorders, alleviate certain eye conditions, and combat inflammation, among various functions [45]. PC (20:5) and TG (20:5) containing EPA, as highlighted by Zhang et al. [46], are noted for their potential to enhance brain function, exert antitumor effects, and reduce plasma lipid concentrations, obesity risk, and cancer incidence via the daily dietary n-3/n-6 ratio. Following criteria such as VIP > 1, FDR < 0.05, and FC > 2 or <0.5, Hou et al. [47] identified significantly different components such as PC, PE, PI, CL, and TG. Ma et al. [48] used volcano plots (FC > 2 or <0.5) with VIP > 1 and FDR < 0.01 to identify and filter differential lipids. For additional correlation analysis, 29 lipids were subsequently retained, 8 of which were downregulated and 21 of which were upregulated. These results suggest that the most different molecular composition of UFA-rich PC is more abundant in low IMF. TGs and phospholipids, owing to their high proportions of UFAs (which are more susceptible to lipid oxidation), contribute to the scent and flavor of meat [49].

Among the 22 differential lipids, 1 was FA, 7 were GL, 10 were GP, and 4 were SP, FA, or TG, which typically refer to esters formed by glycerol and fatty acids (encompassing saturated and unsaturated fatty acids). GP is the most abundant type of phospholipid; it is a crucial element of the cell membrane bilayer structure and is involved in membrane recognition and signal transduction. SPs are polar lipids, the second-largest class of membrane lipids after phospholipids [15]. These findings suggest that the lipid profiles of giant salamander tails differ between sexes, potentially influencing their odor traits.

### 3.2. Qualitative Detection of VOCs via GC-IMS

**Figure 4:**
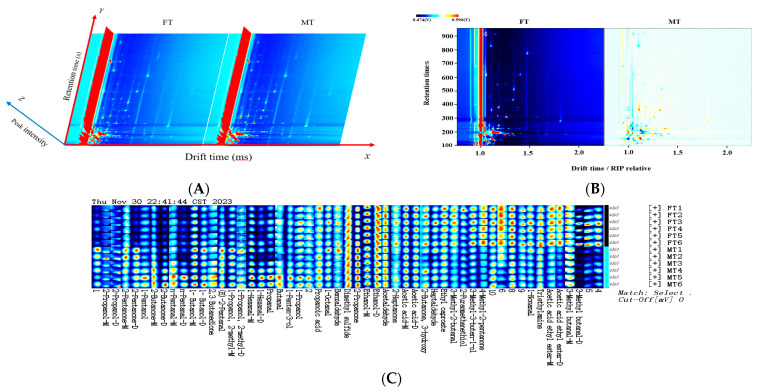
GC-IMS spectra of giant salamander tails of different sexes. Three-dimensional view (**A**); two-dimensional view (**B**). Fingerprint map (vertical axis represents volatile components, whereas the horizontal axis displays giant salamander tail samples of different sexes) (**C**). M stands for monomer, D stands for dimer, FT stands for female tails, and MT stands for male tails. The color represents the content of volatile compounds.

The instrumental 3D spectra of the giant salamander tails via GC-IMS are illustrated in Figure 4A. The differences in the 2D comparison are shown in Figure 4B. The volatile components exhibited distinct separation in the gas phase ion mobility spectra, characterizing the differences in the quantity of certain volatile components between the female tail (FT) and female tail (MT) groups. Figure 4C presents a qualitative analysis of volatile components in giant salamander tails, taking the female tail group as an example. The numbers and labeled points in the figure represent specific volatile components identified through qualitative analysis. Using the retention index database and the migration time database integrated into the software, a total of 45 volatile compounds were detected (Table S2), incorporating 14 alcohols, 1 ether, 14 aldehydes, 3 acids, 9 ketones, 3 esters, and 1 amine. As shown in Table S2, the most abundant compound was 2-propanone, followed by ethanol and acetic acid. The relative content of 2-propanone in the male tails of giant salamanders was markedly greater than that in the female tails (FTs) (*p* < 0.05), whereas the proportions of ethanol and acetic acid were greater in the female tails of giant salamanders than in the male tails (*p* < 0.05). A previous report also identified 2-propanone and acetic acid in different edible parts of giant salamanders on the basis of the GC-IMS approach [10], but no ethanol was detected, which was different from the present results, perhaps because of sample pretreatment procedures and instrumental conditions.

### 3.3. Fingerprint Profiles of Giant Salamander Tails of Different Sexes

The alterations in volatile compounds in giant salamander tails were analyzed via GC-IMS, and the obtained flavor fingerprint profiles are shown in Figure 4C. Since high concentrations of monomer ions and neutral molecules may form dimers in the drift region, a single compound may produce multiple signals, such as a monomer (M) and a dimer (D) [50]. The color of the points for volatile compounds in the fingerprint profiles denotes the relative change. The color represents the content of volatile compounds; the brighter the color is, the higher the content is, indicating differences in volatile flavors among the different groups. The vertical axis displays the same volatile compound components in the giant salamander tails, whereas the horizontal axis shows the giant salamander tails (from top to bottom: female groups and male groups, each with six parallel samples). The red signal indicates a relatively high substance content. For certain substances, there are evident red–blue contrasts between different sexes. For example, in the female tail group, acetic acid ethyl ester-D, acetic acid ethyl ester-M, 4-methyl-2-pentanone, and 3-methyl-3-buten-1-ol had significantly higher levels. Conversely, in the male tail group, substances such as 2-propanone, 1-propanol, 2-methyl-M, (E)-2-pentenal, 2,3-butanedione, n-pentanal, 2-pentanone, and 2-propanol had relatively high levels. Some substances did not significantly differ between sexes, suggesting that giant salamander tails may exhibit distinct flavor characteristics, possibly influenced by differences in lipid and protein compositions. Additionally, previous research has indicated that dimethyl sulfide, 1-nonanal, and trimethylamine are the main contributors to the fishy odor of aquatic products [51]. According to the fingerprint profiles, the levels of these substances were relatively high in each group, indicating that the presence of these compounds in the giant salamander tails significantly contributed to their flavor profile. Previous studies utilized GC-IMS technology to examine volatile flavor compounds in *Crassostrea gigas* during high-temperature cultivation, and 54 volatile elements were detected. Differences in volatile substances between different sexes have been successfully characterized [50]. Moreover, flavor and odor also depend on diet, not only on sex but also on dietary lipids [52]. Guo et al. [53] proposed that dietary fatty acid composition can affect fatty acid composition in animal muscles and that the giant salamander is capable of converting dietary alpha-linolenic acid (ALA) and linoleic acid (LA) into long-chain polyunsaturated fatty acids (LC-PUFAs), which can affect the flavor of the salamander.

To visualize the discrepancies in volatile organic compounds found in giant salamander tails, the peak intensities of odor compounds in the gallery dactylograms were standardized to derive the relative proportions of volatile components in the tails. As shown in Figure S2, the volatile components in giant salamander tails can be broadly categorized into seven compound classes, namely, alcohols (29.96~34.85%), ethers (1.25~1.41%), aldehydes (21.07~22.75%), acids (5.74~6.66%), ketones (30.83~39.57%), esters (1.43~2.17%), and amines (0.98~1.34%). Figure S2 also shows that the ketone content is greater in the male tail group, the alcohol content is greater in the female tail group, and there are subtle differences in other volatile compound categories between different sexes, which might be sex-specific odors [25,26].

### 3.4. Multivariate Statistical Calculation of Tail Vodor Substances of Different Sexes

**Figure 5:**
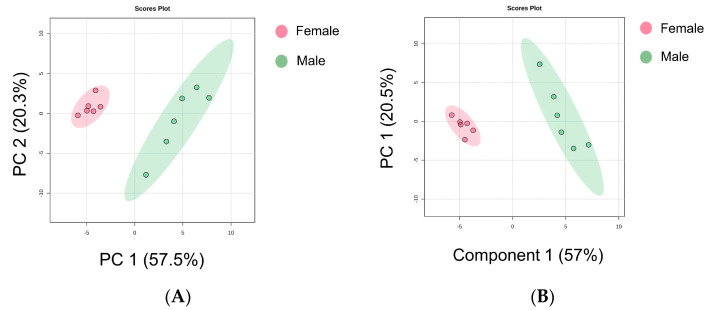

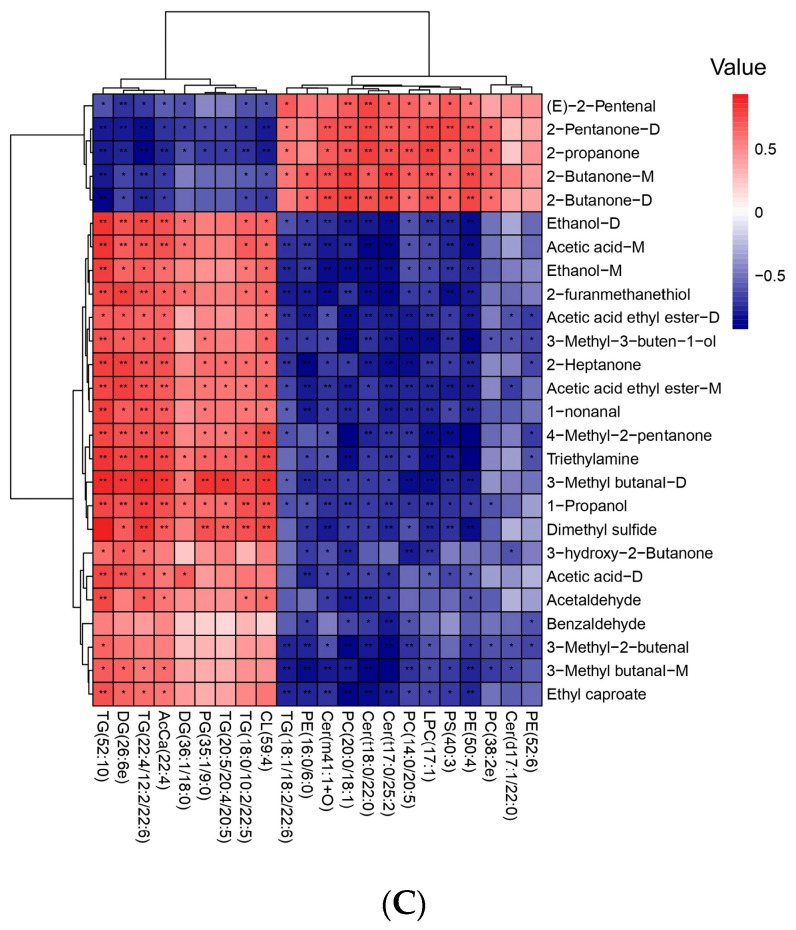
PCA score plot (**A**). PLS-DA score plot (**B**). Heatmap showing the correlation between characteristic volatile compounds and differential lipids, and the asterisks denote statistical significance (* *p* < 0.05, ** *p* < 0.01) (**C**). M stands for monomer and D stands for dimer.

Through the PCA model, we identified discrete differences between volatile compound data points in giant salamander tails, as shown in Figure 5A. PC1 contributed 57.5%, PC2 contributed 20.3%, and the sum of the contributions of the two principal components was 77.8%. A distinct segregation trend was observed between the two groups. Furthermore, we employed PLS-DA, wherein the total contribution rate was 77.5%, and there was also deterministic separation (Figure 5B). The PLS-DA cross-validation results showed *R*^2^ = 0.94594 and *Q*^2^ = 0.88495, indicating the model’s robust classification predictive ability and stability. Sun et al. [50] employed the PCA method to distinguish volatile organic compounds in *Crassostrea gigas* of different ploidies and sexes. On the basis of the degree of sample clustering and dispersion, significant characteristic differences were observed among the different ploidies and sexes. Therefore, the combination of GC-IMS and PCA may better differentiate the polymorphism and sex of volatile organic compounds in oysters. In a study by Jin et al. [14], the PLS-DA method was utilized to screen 13 characteristic volatile substances during the adulteration process of giant salamander oil, and each sample group exhibited clear differentiation, indicating a satisfactory classification effect of the model.

### 3.5. Selection of Characteristic Volatile Components in Giant Salamander Tails of Different Sexes

By employing PLS-DA analysis and filtering criteria with VIP ≥ 1 and *p* < 0.05, a cumulative of 26 characteristic odor compounds were chosen, as outlined in Table 1. These included acetic acid ethyl ester, 4-methyl-2-pentanone, 2-pentanone-D, 3-methyl-2-butenal, 3-methyl butanal-D, triethylamine, 2-propanone 3-methyl butanal-M, ethanol-D, 2-heptanone, 3-methyl-3-buten-1-ol, acetic acid-M, 2-butanone-M, ethanol-M, 1-propanol, ethyl caproate, (E)-2-pentenal, 1-nonanal, dimethyl sulfide, 2-furanmethanethiol, 3-hydroxy-2-butanone, acetic acid-D, 2-butanone-D, acetaldehyde, and benzaldehyde. Different types of compounds have different aroma characteristics. Aldehydes are the primary products of fat degradation, with low thresholds and significant contributions to the overall flavor of giant salamander tails. The fishy odor in aquatic products originates from farming, processing, and storage. During storage, lipid oxidation produces aldehydes such as 3-methyl-2-butenal, 3-methyl butanal-D, 3-methyl butanal-M, (E)-2-pentenal, 1-nonanal, and acetaldehyde, which can lead to the development of a fishy smell in giant salamander tails [42]. Ketones and alcohol are derived mainly from fat oxidation, with thresholds higher than those of aldehydes. Table 1 shows that the content of 2-propanone was the highest. A similar study on *Crassostrea gigas* detected 2-propanone, which is a ketone [30]. Ketones play important roles in the fragrance and fruity flavor of aquatic products because of their high concentration and low threshold. In addition, the relative contents of 3-methyl butanal, ethanol, acetic acid, and acetaldehyde are relatively higher than those of other compounds, and these compounds have higher concentrations in both the female and male tail groups, contributing significantly to the meaty flavor. Research has indicated that 3-methyl butanal, which arises from the oxidation of fatty acids, possesses distinctive odor properties, including a caramel-like flavor [54]. The overall odor of giant salamander tails is formed through the types, concentrations, and interactions of these compounds, which collectively determine the unique odor characteristics of giant salamander tails. A study of *Crassostrea gigas* also revealed differences in volatile compounds between sexes, suggesting that these differences may be related to the distinct nutritional needs of eggs and sperm, leading to varying concentrations in the male and female gonads. Furthermore, the formation of volatile flavors is related to chemical reactions involving lipids, proteins, and sugars [30].

### 3.6. Correlation Assay between Fat and Odor Constituents

To gain deeper insights into how fat degradation and oxidation impact the generation of odor substances, we selected characteristic lipid metabolites and differential volatile compounds for correlation analysis. The results, depicted in Figure 5C, display positive correlations in red and negative correlations in blue. The intensity of the color reflects the strength of the correlation, and vice versa. As shown in Figure 5C, the concentrations of some volatile compounds were positively correlated with the concentrations of the corresponding lipid components. Conversely, other volatile compounds were negatively correlated with the corresponding lipids. For example, the concentrations of triethylamine, dimethyl sulfide, ethanol-D, and 3-methyl butanal-D are positively correlated with lipids such as DG (26:6e), CL (59:4), AcCa (22:4), and TG (52:10) but clearly negatively correlated with lipids such as PC (20:0/18:1), Cer (t18:0/22:0), Cer (t17:0/25:2), and PE (50:4). The levels of compounds such as 2-butanone, 2-pentanone, 2-propanone, and (E)-2-pentenal were significantly negatively correlated with the levels of lipids such as DG (26:6e), CL (59:4), AcCa (22:4), and TG (52:10), but the levels of these compounds were clearly positively correlated with the levels of lipids such as PC (20:0/18:1), Cer (t18:0/22:0), Cer (t17:0/25:2), and PE (50:4). Several studies have indicated that UFAs are major players in the flavor of meat products, as UFAs contain double bonds that are easily oxidized under heating conditions, affecting the flavor of meat products [55].

In giant salamander tails, the oxidation of UFAs easily results in the formation of some fishy odor compounds, including dimethyl-sulfide, 1-nonanal, and trimethylamine, which are low-threshold volatile compounds that contribute to the fishy odor of giant salamander tails [51]. The biochemical reactions accompanying the death of aquatic animals increase the levels of these compounds, subsequently reducing consumer purchase intentions. Figure 5C also shows a negative correlation between these fishy odor compounds and certain lipids, such as PC (20:0/18:1) and LPC (17:1), whereas a positive correlation was observed with TG (52:10) and TG (22:4/12:2/22:6). Tian et al. [31] investigated the relationship between the aroma characteristics and lipid profiles of abalone (*Haliotis discus hannai*). They reported that carbonyl compounds (aldehydes and ketones) and alcohols were positively correlated with phospholipids but negatively correlated with fatty acids, which is similar to our observed results. The results of Zheng et al. [15] also confirmed the correlation analysis of differential lipid components and characteristic odor substances in hams, indicating that the majority of ketone compounds in Nuodeng ham were formed primarily through reactions associated with UFAs.

### 3.7. Use of Differential Lipids and Volatile Compounds for Sex Prediction via Random Forest

Although the present study revealed differential lipids and volatile compounds in the giant salamander tails of different sexes, these differences could still be investigated for further sex prediction. First, the PCA score plots of differential lipids and differential volatile compounds in giant salamander tails of different sexes were generated. As shown in Figure S3, the 22 differential lipids can favorably discriminate female tails from male tails, and the sum ratio of the first two principal components was 72.1%. Moreover, the 26 differential volatile compounds could also be used to classify female tails and male tails clearly, as the sum ratio of the first two components reached 78.4% (Figure S4). These results suggest that the 22 differential lipids and 26 differential volatile compounds represent the majority of the lipid profiles and flavor volatiles in giant salamander tails.

**Figure 6:**
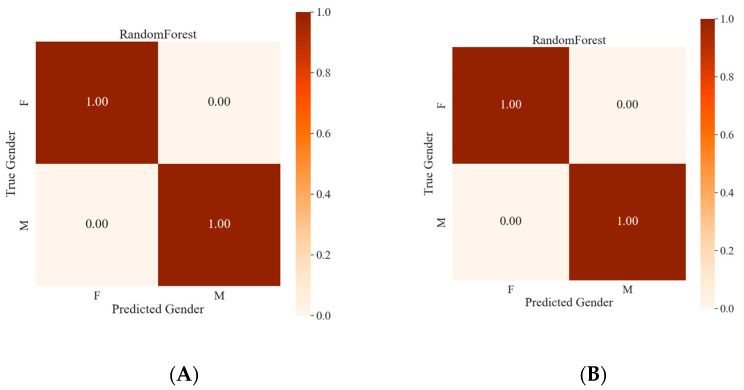
The performance metrics of the random forest model for the 22 differential lipids (**A**) and 26 characteristic volatile flavor compounds (**B**) through threefold cross-validation.

Colón-Crespo et al. [56] noted that marker volatile compounds could be used for the classification of individuals by sex. Frazier et al. [57] reported that lipid biomarkers were associated with age (young and elderly) and sex (male and female). The performance metrics of the random forest model, which uses 22 differential lipids and 26 characteristic volatile compounds for sex prediction, are shown in Figure 6. By threefold cross-validation, the accuracy of 22 differential lipids (Figure 6A) and the accuracy of 26 characteristic volatile compounds (Figure 6B) based on the random forest model were both 100%, implying that they could be used for sex recognition of giant salamanders. We also compared the differences in lipid components and odor substances in the livers of giant salamanders of different sexes. In a recent study, gender prediction with high accuracy based on differential lipids and volatile compounds in giant salamander livers via a random forest model was also reported [33]. Zheng et al. [58] proved that a random forest could diminish overfitting and had the best classification capacity, as it introduced randomness, possessed good noise “immunity” and was suitable for prediction. Even though the present random forest approach possessed perfect accuracy for classifying giant salamander sex, it should still be validated through a larger sample size in the future.

## 4. Conclusions

This study utilized UHPLC-QE-MS and GC-IMS methodologies to identify distinct lipids and volatile organic compounds in giant salamander tails of different sexes. A total of 3145 lipids were identified, with TGs, PCs, and PEs being the predominant categories. Comparative analysis revealed 57 upregulated and 48 downregulated lipids in female tails compared with male tails, with 22 key differential lipids identified through multivariate statistical analysis. Additionally, 45 volatile components were detected, with 26 specific volatile flavor compounds selected via similar statistical methods. Correlation analysis revealed positive associations between the concentrations of certain volatile compounds and specific lipid species, such as triethylamine and dimethyl sulfide, with DG (26:6e) and CL (59:4), respectively. Moreover, 2-butanone and (E)-2-pentenal were positively correlated with PC (20:0/18:1) and Cer (t17:0/25:2), respectively. Both accuracies of these differential lipids and volatiles for sex prediction reached 100%. These findings offer valuable insights into the lipid profiles and flavor characteristics of giant salamander tails across sexes.

## Figures and Tables

**Table 1:** Differential volatile compounds in giant salamander tails of different sexes.

Compounds	VIP	Peak Intensity/mV	
		FT	MT
Acetic acid ethyl ester-D	1.3438	84.83 ± 8.11 ^a^	37.48 ± 2.46 ^b^
Acetic acid ethyl ester-M	1.3331	202.86 ± 5.21 ^a^	145.50 ± 5.10 ^b^
4-Methyl-2-pentanone	1.3296	166.02 ± 10.77 ^a^	102.24 ± 5.69 ^b^
2-Pentanone-D	1.3278	45.83 ± 8.44 ^b^	161.24 ± 15.42 ^a^
3-Methyl-2-butenal	1.3014	71.18 ± 6.66 ^a^	53.43 ± 2.62 ^b^
3-Methyl butanal-D	1.2969	219.64 ± 54.76 ^a^	64.19 ± 11.68 ^b^
Triethylamine	1.2756	350.08 ± 15.69 ^a^	294.14 ± 10.84 ^b^
2-propanone	1.2708	5721.26 ± 174.14 ^b^	7974.35 ± 128.34 ^a^
3-Methyl butanal-M	1.2615	1536.90 ± 57.66 ^a^	1391.87 ± 46.38 ^b^
Ethanol-D	1.2416	4736.03 ± 58.43 ^a^	4316.79 ± 96.18 ^b^
2-Heptanone	1.2217	73.46 ± 5.62 ^a^	66.04 ± 3.77 ^b^
3-Methyl-3-buten-1-ol	1.2082	125.12 ± 8.17 ^a^	92.73 ± 10.91 ^b^
Acetic acid-M	1.2022	1485.14 ± 30.31 ^a^	1446.05 ± 19.75 ^b^
2-Butanone-M	1.1924	460.89 ± 25.70 ^b^	746.61 ± 119.73 ^a^
Ethanol-M	1.1776	2214.83 ± 22.18 ^b^	2216.98 ± 7.55 ^a^
1-Propanol	1.1691	78.08 ± 5.36 ^a^	75.12 ± 5.45 ^b^
Ethyl caproate	1.1596	279.36 ± 12.80 ^a^	244.49 ± 15.14 ^b^
(E)-2-Pentenal	1.1553	333.29 ± 37.73 ^b^	545.13 ± 49.62 ^a^
1-nonanal	1.1422	363.31 ± 39.39 ^a^	282.66 ± 34.66 ^b^
Dimethyl sulfide	1.1317	368.73 ± 6.93 ^b^	373.81 ± 8.37 ^a^
2-furanmethanethiol	1.1253	267.07 ± 39.75 ^a^	220.83 ± 10.01 ^b^
3-hydroxy-2-Butanone	1.1045	447.59 ± 43.65 ^a^	390.86 ± 56.48 ^b^
Acetic acid-D	1.0873	142.20 ± 7.58 ^a^	140.73 ± 9.82 ^b^
2-Butanone-D	1.078	739.44 ± 58.36 ^b^	1926.4 ± 858.88 ^a^
Acetaldehyde	1.0737	1407.58 ± 89.54 ^a^	1374.02 ± 68.01 ^b^
Benzaldehyde	1.0431	63.02 ± 4.52 ^a^	57.47 ± 6.34 ^b^

Different lowercase letters (a, b) in the same row indicate significant differences (*p* < 0.05).

## Data Availability

The original contributions presented in the study are included in the article/supplementary material, further inquiries can be directed to the corresponding author.

## Supplementary Materials

The following supporting information can be downloaded at: https://www.mdpi.com/article/10.3390/foods13193048/s1, Figure S1: Comparison of EPA and DHA in giant salamander tails of different sexes; Figure S2: Relative content of volatile categories in female and male tails of giant salamander; Figure S3: PCA score plot of 22 differential lipids in giant salamander tails of different sexes; Figure S4: PCA score plot of 26 differential volatile compounds in giant salamander tails of different sexes; Table S1: Differential lipids in female and male tails of giant salamander; Table S2: Volatile organic compounds identified in female and male tails of giant salamander.
