# Supplementary material for: Sex-Specific Lipid Profiles and Flavor Volatiles in Giant Salamander (Andrias davidianus) Tails Revealed by Lipidomics and GC-IMS
Source: Foods. 2024 Sep 25;13(19):3048. doi: 10.3390/foods13193048 (PMC11476126; doi:10.3390/foods13193048)
Supplement: Supplementary file 1 [file foods-13-03048-s001.zip › foods-3200415-supplementary.pdf]

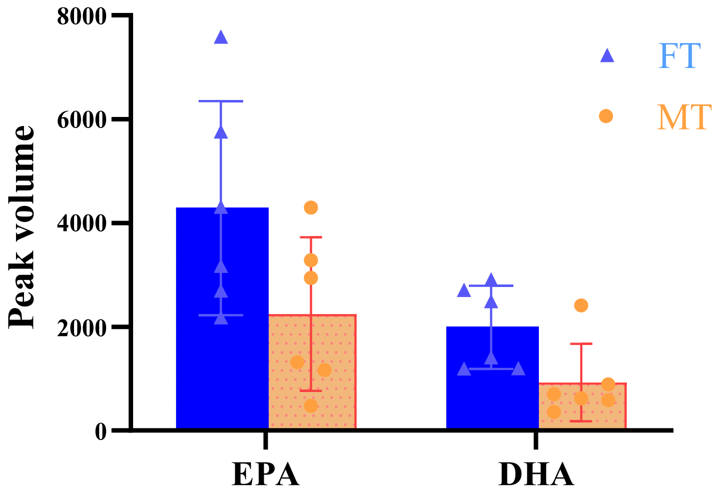

Figure S1. Comparison of EPA and DHA in giant salamander tails of different sexes.

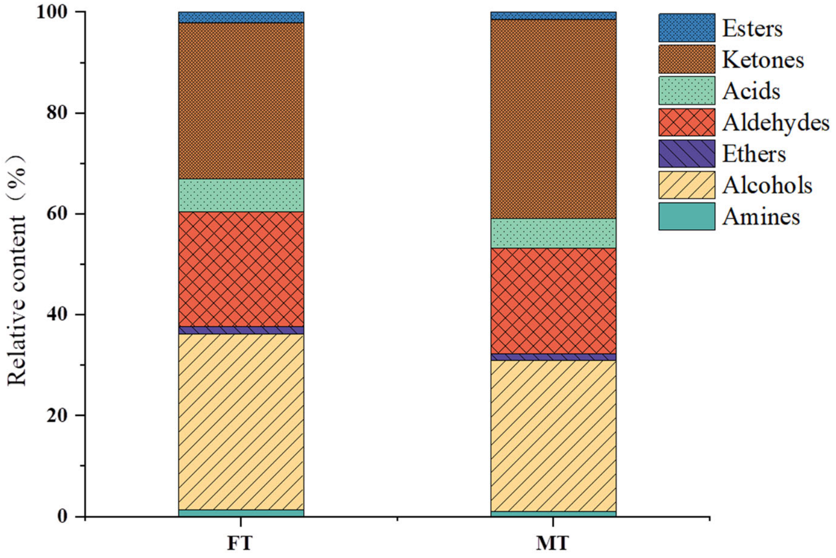

Figure S2. Relative content of volatile categories in female and male tails of giant salamander.

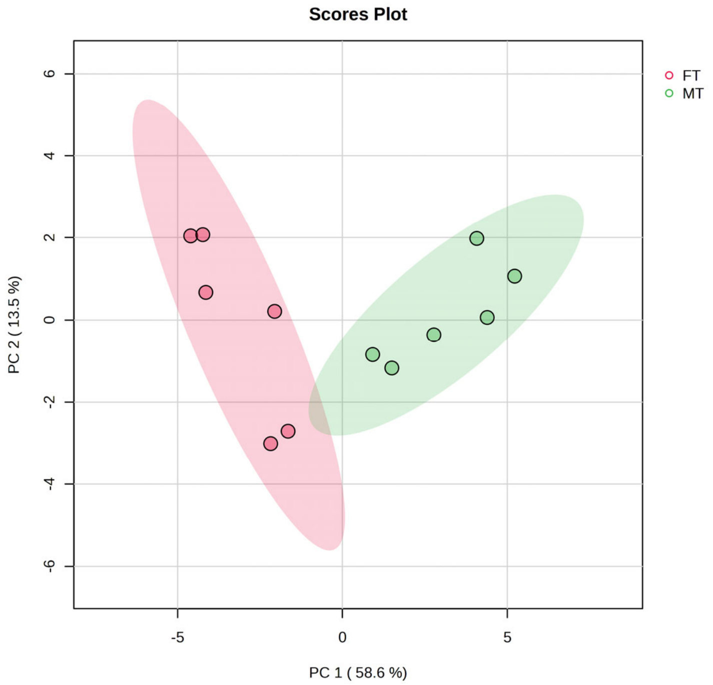

Figure S3. PCA score plot of 22 differential lipids in giant salamander tails of different sexes.

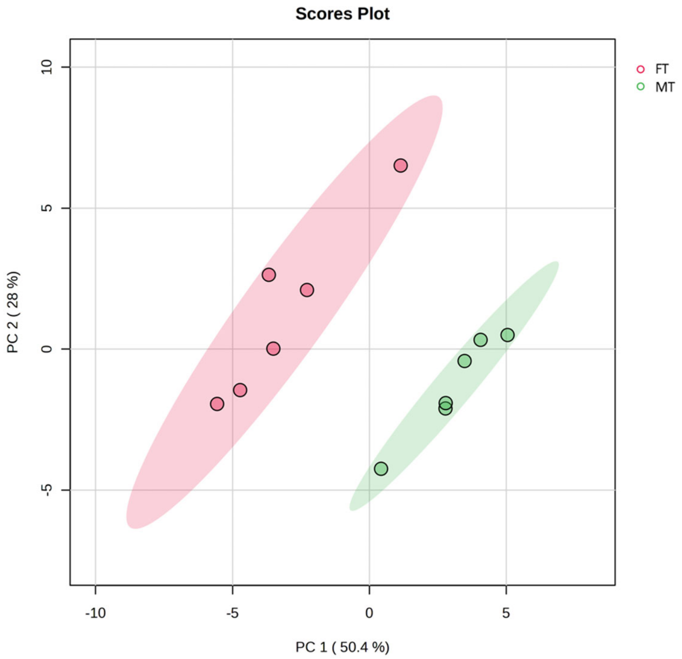

Figure S4. PCA score plot of 26 differential volatile compounds in giant salamander tails of different sexes.

Table S1 Differential lipids in female and male tails of giant salamander.

name	FC	VIP	FT	MT
LPC(20:3e)+H	0.0071665	1.5426	0.00±0.00	0.34±0.76
PI(29:0/18:1)+H	0.022868	2.1207	1.57±1.43	19.92±25.27
PC(14:0/20:5)+Na	0.03085	1.826	0.00±0.00	0.03±0.01
PS(40:3)+H	0.038647	1.8344	0.00±0.00	0.03±0.02
PC(36:3)+H	0.046155	1.3734	0.00±0.00	0.03±0.02
TG(18:1/18:2/22:6)+H	0.048338	1.3039	0.01±0.00	0.04±0.05
TG(45:12)+NH4	0.059258	1.3379	0.03±0.02	0.15±0.16
TG(18:1/20:5/22:5)+H	0.059374	1.8753	0.03±0.02	0.16±0.13
LPC(16:1e)+H	0.063079	3.7029	0.01±0.03	0.07±0.04
Cer(m41:1+O)+Na	0.065332	1.1945	0.00±0.00	0.01±0.00
LPC(20:4e)+H	0.070132	1.8993	0.00±0.00	0.02±0.01
PE(16:0/6:0)-H	0.076705	1.2486	0.01±0.00	0.02±0.01
Hex2Cer(d32:3)+HCOO	0.077009	2.7942	0.05±0.06	0.32±0.28
PC(38:2e)+H	0.07837	1.6527	0.06±0.06	0.38±0.30
PC(20:0/18:1)+HCOO	0.082511	1.2382	0.04±0.03	0.15±0.01
ST(m20:1/25:2)+NH4	0.084362	1.3065	0.01±0.01	0.04±0.02
TG(14:0e/10:3/18:2)+NH4	0.087079	1.1734	0.01±0.00	0.04±0.02
PI(47:0)+NH4	0.088452	1.7925	1.49±1.13	8.61±7.77
Cer(t18:0/22:0)+HCOO	0.093787	1.1092	0.01±0.00	0.02±0.01
PC(31:2)+H	0.10013	1.0094	0.02±0.01	0.07±0.06
PC(18:0/18:0)+HCOO	0.10108	1.0486	0.03±0.02	0.13±0.06
LPC(17:1)+H	0.10552	1.5976	0.01±0.00	0.04±0.04
PC(18:1/14:2)+HCOO	0.10619	4.3238	0.42±0.52	2.19±0.85
ST(d40:0+O)+NH4	0.10764	1.0279	0.00±0.00	0.00±0.00
Cer(d17:1/22:0)+HCOO	0.11137	1.043	0.04±0.04	0.08±0.02
DG(8:0/20:3)+NH4	0.11211	1.2048	1.12±0.91	4.36±1.98
PC(42:6e)+H	0.11617	1.1255	0.01±0.01	0.01±0.01
PE(50:4)+Na	0.11647	1.6765	0.05±0.04	0.24±0.09
Hex2Cer(t42:2)+HCOO	0.12808	1.114	0.01±0.01	0.03±0.02
Cer(t17:0/25:2)+HCOO	0.13008	1.0227	0.02±0.01	0.05±0.01
TG(18:0/13:0/22:6)+NH4	0.14136	1.327	2.61±3.52	6.56±4.34
DG(36:6e)+Na	0.14597	1.6628	3.43±4.40	10.67±6.21
PE(52:5)+H	0.14917	1.2777	0.36±0.34	1.24±0.59
Cer(d18:1/24:0)+HCOO	0.15966	1.2994	0.09±0.08	0.17±0.02
PI(43:0)+Na	0.16404	1.1132	3.17±2.15	12.25±11.63
DG(37:3)+NH4	0.18028	1.2811	2.57±2.55	7.88±3.97
ST(d50:0)+H-H2O	0.18073	1.1613	0.02±0.02	0.07±0.02
PI(47:1)+H	0.19552	1.0068	1.51±1.10	3.47±1.23
PE(52:6)+Na	0.20287	1.3925	0.02±0.02	0.08±0.05
PC(32:4e)+HCOO	0.20415	1.4268	0.26±0.22	0.61±0.19
Cer(t38:3)+H	0.20922	1.0744	0.04±0.03	0.10±0.03
TG(12:0e/11:3/20:4)+NH4	0.22002	1.061	0.00±0.00	0.01±0.00

ZyE(36:2)+H	0.22193	5.3023	23.48±30.61	23.74±17.95
PC(8:1e/14:4)+Na	0.22369	1.4061	0.01±0.01	0.02±0.01
LPC(20:2e)+H	0.23447	2.4494	0.05±0.11	0.17±0.19
PI(34:5)+NH4	0.24602	1.5752	5.46±5.54	11.46±4.07
LPI(18:3e)+Na	0.25684	1.6908	4.50±4.45	7.80±1.34
PE(54:7)+H	0.41964	1.3058	0.90±1.38	2.10±1.78
TG(54:11)+NH4	2.0399	1.591	0.09±0.07	0.01±0.01
TG(18:0e/16:0/20:4)+H	2.054	1.3925	0.12±0.08	0.02±0.02
PG(30:1/16:0)+H	2.0819	1.3011	0.46±0.42	0.10±0.09
SM(d42:1)+H	2.1265	1.4566	0.23±0.18	0.03±0.01
SM(d20:1/24:0)+H	2.3214	1.4021	2.73±2.29	0.43±0.30
TG(11:0/18:2/18:3)+NH4	2.3455	1.6078	2.59±2.10	0.56±0.46
DG(36:1/16:0)+NH4	2.398	1.3537	85.6±77.2	11.44±5.08
PE(18:0/22:5)-H	2.4681	1.8266	4.08±3.17	1.12±1.93
FA(22:6)-H	2.4729	1.5797	5.06±3.82	0.90±1.06
AcCa(17:0)+H	2.5403	1.6192	0.19±0.13	0.03±0.03
DG(36:1/18:1)+NH4	2.5534	1.3914	78.66±78.06	8.60±2.83
PE(16:1e/18:2)+Na	2.5833	2.1269	7.66±4.29	3.17±6.38
PG(27:1/21:1)+H	2.6136	1.3972	1.88±1.94	0.20±0.11
TG(14:0/14:0/22:6)+NH4	2.7035	1.6083	39.23±27.83	5.36±5.70
TG(18:0/10:2/22:5)+NH4	2.7633	1.5586	0.08±0.05	0.02±0.02
FA(22:4)-H	2.7677	1.7798	0.36±0.21	0.04±0.02
PC(21:0/14:2)+Na	2.8362	1.8794	1.34±1.24	0.32±0.55
PC(15:0/22:5)+H	2.844	1.8786	1.34±1.25	0.32±0.55
PC(38:7e)+H	2.8764	2.2774	60.89±51.67	15.25±29.4
PC(34:4e)+HCOO	2.9206	1.8601	0.84±0.45	0.19±0.27
PE(16:1p/20:4)+H	2.9206	2.0038	13.05±7.08	3.06±4.32
TG(18:3/14:4/20:5)+NH4	2.9364	1.6891	0.06±0.04	0.01±0.00
PG(29:1/20:2)+NH4	2.9488	1.5392	0.6±0.63	0.07±0.07
TG(16:0e/16:0/18:1)+NH4	2.9686	1.5937	1.07±0.91	0.17±0.20
PG(20:0/22:6)+NH4	2.9943	1.9239	0.04±0.05	0.00±0.00
TG(18:1e/16:0/18:1)+NH4	3.0853	1.6507	4.23±3.65	0.64±0.83
Hex1Cer(d40:1)+HCOO	3.1136	1.6396	0.13±0.08	0.04±0.06
PC(18:2e/22:5)+HCOO	3.1924	2.609	3.80±2.37	1.01±1.44
TG(20:5/20:4/20:5)+NH4	3.2875	1.5576	0.11±0.09	0.01±0.01
PE(39:5e)+H	3.3301	2.1177	136.06±96.72	27.28±47.06
TG(52:10)+NH4	3.3318	1.898	0.12±0.08	0.01±0.01
PG(36:3e)-H	3.4364	1.6385	0.21±0.04	0.03±0.01
PI(18:0/22:4)-H	3.506	2.1097	2.60±1.46	0.30±0.40
PG(16:0/18:1)-H	3.5254	1.7853	19.46±13.64	3.14±4.42
PC(16:0e/22:5)+Na	3.5703	2.0196	0.66±0.42	0.12±0.20
TG(18:0e/16:0/18:1)+NH4	3.7047	1.9004	1.23±1.23	0.01±0.13
PC(16:1e/20:4)+HCOO	3.7716	2.4873	58.26±50.54	10.93±20.78
LPI(35:0)-H	3.8014	1.9678	49.18±44.76	3.57±3.07

PC(18:2e/20:4)+HCOO	3.8273	2.2479	54.33±40.47	9.42±16.35
CL(59:4)-2H	3.9433	1.7319	0.13±0.06	0.03±0.03
PG(46:2)+H	3.9819	1.6752	5.68±5.78	0.44±0.27
PE(18:0e/22:6)+H	4.0527	2.5532	10.66±7.49	1.97±3.53
PS(38:2)+Na	4.2835	2.1137	0.04±0.03	0.00±0.00
AcCa(22:4)+H	4.3831	2.2111	0.09±0.05	0.01±0.02
PG(35:1/9:0)+H	4.4754	1.785	2.33±2.52	0.17±0.12
TG(18:1/14:0/22:6)+H	4.628	1.99	0.31±0.31	0.03±0.04
DG(26:6e)+NH4	4.7938	2.1194	6.05±1.86	0.95±1.09
Hex1Cer(d36:1)+HCOO	5.1143	1.8257	0.45±0.25	0.06±0.08
DG(36:1/18:0)+NH4	5.3546	1.8217	12.88±12.42	0.89±0.62
PI(18:0/16:1)-H	5.3997	2.6896	10.08±6.85	0.85±0.98
TG(18:0/18:0/18:0)+Na	5.4847	2.2457	0.34±0.23	0.05±0.08
TG(22:4/12:2/22:6)+NH4	5.518	1.9833	0.24±0.22	0.02±0.02
PG(48:3)+H	5.9367	1.87	4.21±5.20	0.23±0.20
TG(18:0e/18:1/22:4)+NH4	7.4081	2.2658	0.02±0.02	0.00±0.00
LPC(14:1e)+HCOO	8.5566	7.2714	0.00±0.00	0.00±0.00
TG(20:0e/16:0/18:1)+NH4	10.512	2.4702	0.54±0.76	0.02±0.04
CL(79:10)-2H	147.86	4.6048	49.26±81.8	0.08±0.07

Note: differential lipids were selected using criteria of VIP ≥ 1, $P < 0.05$, fold-change (FC) > 2, or < 0.5.

Table S2 Volatile organic compounds identified in female and male tails of giant salamander.

Compounds	VIP	Peak intensity / mV	
		FT	MT
Acetic acid ethyl ester-D	1.3438	84.83±8.11[a]	37.48±2.46[b]
Acetic acid ethyl ester-M	1.3331	202.86±5.21[a]	145.05±5.10[b]
4-Methyl-2-pentanone	1.3296	166.02±10.77[a]	102.24±5.69[b]
2-Pentanone-D	1.3278	45.83±8.44[b]	161.24±15.42[a]
3-Methyl-2-butenal	1.3014	71.18±6.66[a]	53.43±2.62[b]
3-Methyl butanal-D	1.2969	219.64±54.76[a]	64.19±11.68[b]
Triethylamine	1.2756	350.08±15.69[a]	294.14±10.84[b]
2-propanone	1.2708	5721.26±174.14[b]	7974.35±128.34[a]
3-Methyl butanal-M	1.2615	1536.9±57.66[a]	1391.87±46.38[b]
Ethanol-D	1.2416	4736.3±58.43[a]	4316.79±96.18[b]
2-Heptanone	1.2217	73.46±5.62[a]	66.04±3.77[b]
3-Methyl-3-buten-1-ol	1.2082	125.12±8.17[a]	92.73±10.91[b]
Acetic acid-M	1.2022	1485.14±30.31[a]	1446.05±19.75[b]
2-Butanone-M	1.1924	460.89±25.7[b]	746.61±119.73[a]
Ethanol-M	1.1776	2214.83±22.18	2216.98±7.55
1-Propanol	1.1691	78.08±5.36	75.12±5.45
Ethyl caproate	1.1596	279.36±12.8[a]	244.49±15.14[b]
(E)-2-Pentenal	1.1553	333.29±37.73[b]	545.13±49.62[a]
1-nonanal	1.1422	363.31±39.39[a]	282.66±34.66[b]
Dimethyl sulfide	1.1317	368.73±6.93	373.81±8.37
2-furanmethanethiol	1.1253	267.07±39.75[a]	220.83±10.01[b]
3-hydroxy-2-Butanone	1.1045	447.59±43.65	390.86±56.48
Acetic acid-D	1.0873	142.2±7.58	140.73±9.82
2-Butanone-D	1.078	739.44±58.36[b]	1926.4±858.88[a]
Acetaldehyde	1.0737	1407.58±89.54	1374.02±68.01
Benzaldehyde	1.0431	63.02±4.52	57.47±6.34
2-methyl-1-Propanol-M	0.99083	178.5±6.20[b]	245.25±24.98[a]
2,3 Butanedione	0.9825	128.23±16.67[b]	189.97±28.64[a]
n-Pentanal-M	0.92049	193.02±24.58[b]	307.36±70.86[a]
2-Propanol-D	0.86429	166.62±13.23[b]	238.17±30.70[a]
Heptaldehyde	0.76502	281.72±40.51	265.80±46.18
1- butanol-D	0.7257	70.95±7.78[b]	97.55±15.91[a]
Butanal	0.70564	106.17±7.05	102.02±26.65
n-Pentanal-D	0.6271	56.54±11.53	111.21±54.77
1-octanal	0.6269	244.06±26.92	246.58±35.77
1-Pentanol	0.57084	77.91±9.18	110.62±32.78
2-methyl-1-Propanol-D	0.55351	23.74±3.45[b]	30.73±4.20[a]
1-hexanal-D	0.49974	81.82±27.43	137.65±54.51
2-Propanol-M	0.41926	231.87±14.24	247.47±24.09
1- butanol-M	0.30161	560.33±28.26[b]	662.44±52.53[a]

1-hexanal-M	0.26031	743.91±131.55	942.68±229.01
Propanal	0.22508	500.27±119.23	705.92±290.81
1-Penten-3-ol	0.1339	144.75±13.47	164.21±32.36
2-Pentanone-M	0.052109	288.95±13.19	329.74±37.56
Propanoic acid	0.027999	114.43±12.30^{b}	131.07±7.96^{a}

Different lowercase letters (a,b) in the same row indicate significant differences (P <0.05).
